# The Necessity of POMC and MC3R Analysis in the First-Level Diagnosis of Monogenic Obesity: The Experience of Two Italian Centers

**DOI:** 10.3390/genes17040405

**Published:** 2026-03-31

**Authors:** Kateryna Miedviedieva, Tommaso Regoli, Stefania Marchisotta, Luca Sessa, Melania Blasco, Silvana Leanza, Cristina Ciuoli, Anna Cantore, Claudia Ricci, Silvia Cantara

**Affiliations:** 1 Department of Medical, Surgical and Neurological Sciences, University of Siena, 53100 Siena, Italy; kate018535@gmail.com (K.M.); tommaso.regoli@student.unisi.it (T.R.); anna.cantore@unisi.it (A.C.); cantara@unisi.it (S.C.); 2 Simple Operating Unit of Endocrine Surgery, Ospedale “Il Giglio”, 90015 Cefalù, Italy; smarchisotta@gmail.com (S.M.); luca.sessa@hsrgiglio.it (L.S.); 3 Faculty of Medicine, Unicamillus-Saint Camillus International University of Health Sciences, 00131 Rome, Italy; 4 Operating Unit of Internal Medicine, Ospedale “Il Giglio”, 90015 Cefalù, Italy; melania.blasco@hsrgiglio.it; 5 Simple Operating Unit of General Surgery, Ospedale “Il Giglio”, 90015 Cefalù, Italy; silva.leanza@hsrgiglio.it; 6 Simple Operating Departmental Unit of Endocrinology, Ospedale San Donato, 52100 Arezzo, Italy; cristina.ciuoli@uslsudest.toscana.it

**Keywords:** Melanocortin Receptor 3, proopiomelanocortin, obesity, genetic, leptin, lep receptor, brain-derived neurotrophic factor, single-nucleotide polymorphisms

## Abstract

Background/Objectives: Obesity is a global health emergency with a complex etiology, in which monogenic forms, although rare, are significantly underdiagnosed. In our clinical setting, first-tier genetic screening panels targeting *LEP*, *LEPR*, *BDNF*, *FTO*, and *MC4R* often fail to identify a causative variant, leaving a significant diagnostic gap. This study aimed to assess the prevalence of variants in other critical genes of the melanocortin pathway to improve diagnostic yield. Methods: We analyzed 88 patients with non-syndromic obesity (Body Mass Index, BMI > 30 kg/m^2^), who were first screened for our standard obesity-related genes. In those testing negative, we expanded the analysis to include the *MC3R* and *POMC* genes. In silico bioinformatic tools were used to predict the functional consequences of identified variants on protein structure and splicing. Results: We found several variants in *POMC*, specifically within the regions coding for alpha-, beta-, and gamma-MSH peptides. A bioinformatic analysis suggests that these variants disrupt the melanocortin signaling pathway, likely contributing to an intermediate susceptibility phenotype in our adult cohort. Additionally, a clinical follow-up of a patient carrying the rare BDNF p.Thr2Ile variant revealed a suboptimal response to high-dose tirzepatide treatment (9% weight loss over 72 weeks), notably inferior to the average response observed in clinical trials. Conclusions: Our findings demonstrate that expanding first-level routine testing to include *POMC* and *MC3R* is essential to maximize diagnostic yield and improve clinical management.

## 1. Introduction

According to the World Health Organization, 43% of adults were classified as overweight and approximately 16% as obese in 2022. Among children and adolescents aged 5–19, 20% were overweight, highlighting the scale of the problem across all age groups (WHO, Obesity and overweight 2025, available from https://www.who.int/news-room/fact-sheets/detail/obesity-and-overweight, accessed on 25 March 2026). Obesity is a complex multifactorial condition characterized by the excessive accumulation of adipose tissue resulting from a prolonged disruption of energy balance. Its development is driven by a combination of genetic predisposition and environmental influences, and its manifestations may emerge in early childhood and persist across the entire lifespan [1]. Despite numerous family-based linkage scans and genome-wide association studies (GWASs), and the hundreds of associations identified to date, the genetic background of obesity is still not fully elucidated. The genetic basis of energy balance regulation is largely centered on the hypothalamic melanocortin system (MCS), which is critical for controlling food intake and energy expenditure [2]. The pro-opiomelanocortin (*POMC*) gene encodes a precursor polyprotein that is proteolytically cleaved to produce several signaling peptides. These include the potent anorexigenic alpha-melanocyte-stimulating hormone (alpha-MSH), as well as beta-melanocyte-stimulating hormone (beta-MSH) and gamma-melanocyte-stimulating hormone (gamma-MSH) [3,4]. Although alpha-MSH is the primary ligand for Melanocortin Receptor 4 (MC4R), beta-MSH is also a potent MC4R agonist that contributes to the anorexigenic signaling pathway [4]. Gamma-MSH primarily acts on Melanocortin Receptor 3 (MC3R). Although its effect on acute food intake is less pronounced than that of alpha-MSH on MC4R, MC3R is thought to primarily regulate energy partitioning. This influences not only appetite but also determines whether energy is stored as fat or utilized for lean mass [5,6]. Defects in the POMC precursor, which result in the deficiency of all MSH peptides, lead to a severe form of early-onset obesity, often accompanied by red hair and adrenal insufficiency [7]. Similarly, mutations in MC3R disrupt the central signaling cascade of the MCS, leading to hyperphagia and weight gain, thus highlighting the critical importance of all the components of the POMC axis as primary genetic targets in the etiology of obesity [8]. Based on our previous findings [9], we aimed to analyze an additional cohort of patients to further expand the existing knowledge on the genetic factors contributing to obesity. In our clinical setting, we typically evaluate the leptin (*LEP*) and leptin receptor (*LEPR*) genes, as well as the *MC4R* gene, which are associated with monogenic forms of obesity. We also evaluate well-known susceptibility genes, such as the brain-derived neurotrophic factor (*BDNF*) and fat mass and obesity-associated (*FTO*) genes. In the present study, the patients were additionally screened for the *POMC* and *MC3R* genes. This approach allows for a deeper assessment of the genetic background involved in the development of this condition, the identification of new variants, and the discovery of potential genetic targets that may be valuable for designing personalized therapeutic strategies.

## 2. Materials and Methods

### 2.1. Subjects

This study examined 88 adults (mean age 50 years; range 38–67 years), of whom 72.7% (n = 64) were women and 27.3% (n = 24) were men. Of these patients, 21.6% (n = 19) were followed at Ospedale San Donato in Arezzo, Italy, while the remaining 78.4% (n = 69) were followed at Ospedale il Giglio in Cefalù, Italy.

Patients with a body mass index (BMI) > 30 kg/m^2^ (BMI at diagnosis: 46 ± 5.03) who underwent bariatric surgery were included. Initially, obesity was diagnosed based on BMI. Inclusion criteria were BMI ≥ 40 kg/m^2^ or ≥ 35 kg/m^2^ associated with treated or untreated dyslipidemia (cholesterol LDL > 140 mg/dL and/or total cholesterol > 200 mg/dL), hypertension or type 2 diabetes in patients who had not achieved success despite multiple attempts at diet and physical activity interventions. During the medical evaluation, the following parameters were assessed: glycemic control (fasting glucose, insulin and glycated hemoglobin), liver function, and cardiometabolic biomarkers (lipid profile, including triglycerides and LDL/HDL cholesterol, uric acid, pulse rate and blood pressure). To exclude endocrine causes of obesity (such as Cushing’s syndrome), baseline and post-dexamethasone (1 mg) overnight levels of thyroid-stimulating hormone (TSH), free thyroxine (FT4), cortisol and adrenocorticotropic hormone (ACTH) were measured. In our cohort, none of the patients presented with clinical features suggestive of syndromic obesity (such as Prader–Willi, Bardet–Biedl, or Alström syndromes). Furthermore, none of the patients presented with clinical features suggestive of syndromic obesity (e.g., Prader–Willi, Bardet–Biedl, or Alström syndromes) or obesity hypoventilation syndrome (Pickwickian syndrome). Dyslipidemia, steatosis (evaluated using the Fatty Liver Index), HOMA index [Fasting blood glucose (mg/dL) × Fasting insulin (µU/mL)/405] and metabolic syndrome (MS) were identified in the patient group. The MS was determined based on the presence of at least three of the following parameters: abdominal girth, triglycerides, HDL, hypertension, diabetes or fasting glycemia. The clinical features and biological parameters are summarized in Table 1.

Each patient signed informed consent for participation in this study, and the study was approved by the ethical committee (2024/874).

### 2.2. DNA Extraction and PCR

Genomic DNA was extracted from peripheral blood leukocytes using a salting-out technique and stored at −20 °C until PCR analysis. The DNA samples were analyzed by Nanodrop One (Thermo Scientific, Milan, Italy) for concentration and purity. A study of SNPs was conducted by PCR amplification. Specific primers were designed using Primer3 Input tool version 4.1.0 and purchased from Eurofins Genomics (Ebersberg, Germany). Primer sequences and PCR conditions are reported in the Supplementary Materials.

### 2.3. Sequencing

The entire set of samples was directly sequenced by Sanger sequencing using the Thermo Fisher 3500 Series Genetic Analyzer (Thermo Scientific).

### 2.4. Bioinformatic Analyses

The potential pathogenicity of the identified non-synonymous variant was assessed using the PredictSNP consensus classifier, https://loschmidt.chemi.muni.cz/predictsnp/ (accessed on 25 March 2026) [10]. This meta-predictor integrates the outputs of six established prediction tools (SIFT, PolyPhen-1, PolyPhen-2, MAPP, PhD-SNP and SNAP) to generate a unified score. This consensus approach was chosen to improve the overall accuracy and reliability of the pathogenicity prediction. Variants were classified as ‘deleterious’ or ‘neutral’ based on the PredictSNP confidence score. Nature of variants was assessed using ClinVar, https://www.ncbi.nlm.nih.gov/clinvar/ (accessed on 25 March 2026) and Franklin, https://franklin.genoox.com/clinical-db/home (accessed on 25 March 2026).

The protease-specific substrate cleavage prediction in the presence of the identified variants was carried out with the tool ProsperousPlus, http://prosperousplus.unimelb-biotools.cloud.edu.au/index.php/prediction (accessed on 25 March 2026) [11]. Human Splicing Finder Pro, https://genomnis.com/hsf (accessed on 25 March 2026) was used for the splicing prediction.

## 3. Results

### 3.1. Presence of Genetic Variants in the Patient Cohort

In our clinical first-level routine, patients with obesity, suspected of having a monogenic form of the disease, are evaluated for the presence of pathogenetic variants in the *LEP*, *LEPR*, *BDNF*, *FTO*, *MC4R* genes. This specific first-tier panel reflects the standard diagnostic pathways currently driven by local clinical protocols and regional reimbursement guidelines. Consequently, *POMC* and *MC3R* are not typically included in the initial clinician-driven requests. For this reason, we first evaluated this panel in our eighty-eight patients.

No variants were identified in either the *LEP* or *FTO* genes. In the *LEPR* gene, we found three SNPs: the p.Pro1019= (rs1805096) variant was detected in 39 patients (44.3%); the p.Lys109Arg (rs1137100) variant was found in 27 patients (30.7%); and the p.Gln223Arg (rs1137101) variant was present in 18 patients (20.5%). In the *BDNF* gene, the common p.Val66Met variant (rs6265) was identified in 29 patients (33.0%), while the rare p.Thr2lle (rs8192466) variant was found in only one patient (1.1%). In the *MC4R* gene, we identified the p.lle251Leu (rs52820871) variant in two patients (2.3%) and the synonymous variant p.Val103= (rs1278744683) in one patient (1.1%) (see Table 2). As the rs52820871 variant in the *MC4R* gene and the rs8192466 variant in the *BDNF* gene have already been associated with obesity, these patients were excluded from the secondary analysis. In the remaining 85 patients, the analysis was extended to the *POMC* and *MC3R* genes. In the *MC3R* gene the p.Val44lle (rs3827103) missense variant was identified in six patients (7%). An analysis of the *POMC* gene revealed four rare variants, each identified in a single patient (1.2%): the p.Arg90His (rs1216042661); the p.Ser94Gly (rs180767274); the p.Ser94= (rs28930368) and the p.Ala195= (rs2071345). The p.Ser97_Gly99del (rs10654394) insertion variant in POMC was found in 11 patients (12.9%). All the variants were identified in the heterozygous state (see Table 2).

### 3.2. Bioinformatic Analysis of the MC3R Variant

**Figure 1:**
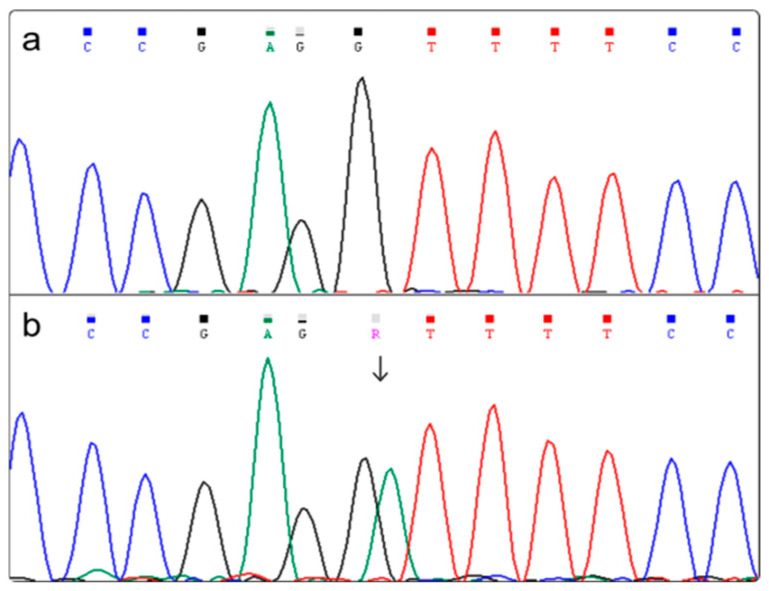
(**a**) Wild-type sequence and (**b**) heterozygous patient for the p.Val44Ile (c.130G>A) in the *MC3R* gene. The arrow indicates the position of the variant in the electropherogram.

The percentage of patients carrying the rs3827103 p.Val44Ile variant in the MC3R gene was consistent with its frequency in the Italian population (see Table 2). All the patients were heterozygous for the variant (Figure 1). The p.Val44Ile substitution involves a change between two chemically similar nonpolar hydrophobic amino acids. For this reason, when analyzed by PredictSNP, it was predicted to be ‘neutral’ with an overall score of 0.74. Conversely, the SIFT program indicated the variant as ‘deleterious’ with a score of 0.43. Franklin classified this variant as benign. The variant does not modify RNA processing by introducing new splice sites (Human Splicing Finder Pro).

### 3.3. Bioinformatic Analysis of the POMC Variants

**Figure 2:**
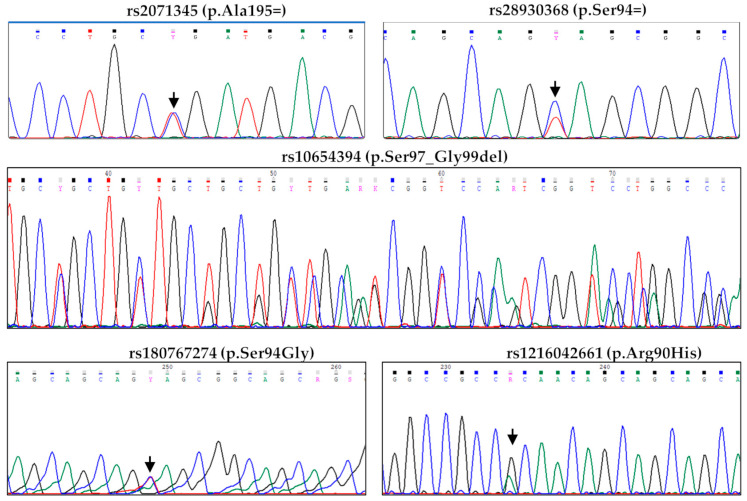
Electropherograms showing *POMC* gene variants. The arrows indicate the position of the variants in the electropherograms.

The *POMC* gene analysis revealed four distinct rare variants: the p.Arg90His, the p.Ser94Gly, the p.Ser94=, and the p.Ala195=. The common variant p.Ser97_Gly99del was also found (Figure 2).

Notably, all of these variants are located within the POMC pro-protein, specifically in regions that are critical for the post-translational processing of active MSH peptides. The p.Arg90His and p.Ser94Gly/Ser94= variants are found in the joining peptide, while the p.Ala195= variant is located within the beta-lipotropin (LPH) fragment, the precursor to beta-MSH.

The PredictSNP analysis scored rs1216042661 p.Arg90His as deleterious (overall score 0.61: PolyPhen-1: 0.74; PolyPhen-2: 0.68; SIFT: 0.79; and SNAP: 0.62). In contrast, rs180767274 p.Ser94Gly was classified as neutral, with an overall score of 0.83. This variant has been reported as having ‘uncertain’ clinical significance in both ClinVar and Franklin.

**Figure 3:**
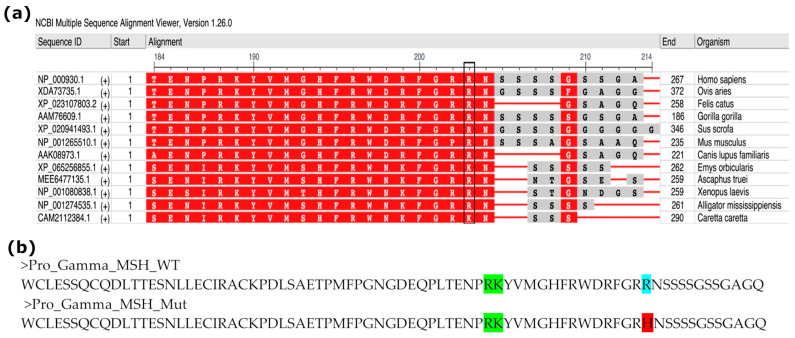
(**a**) BLAST among species of the gamma-MSH sequence. A red background indicates residues that are retained across species. The cleavage site is indicated in the square box. (**b**) Wild-type and mutated sequence of the gamma-MSH. The light blue and red highlight amino acid position 90. Green indicates the proteolytic site upstream.

Regarding the p.Arg90His variant, the critical nature of the dibasic arginine (RR) motif at positions 89–90 for gamma-MSH production has been demonstrated in rodent models, which lack this essential cleavage site by nature. Instead, rodents possess a proline–arginine (PR) motif (Figure 3a, *Mus musculus*) at this locus, which prevents the production of the final gamma-MSH peptide in the pituitary and hypothalamus [12,13,14].

To investigate the impact of the p.Arg90His mutation on peptide processing in humans, we used the ProsperousPlus bioinformatics tool [11] to predict the prohormone convertase (PC) cleavage sites within the pro-gamma-MSH sequence. The in silico analysis identified the wild-type dibasic site (Arg89–Arg90) as a high-probability cleavage site for the PC2 enzyme, with a confidence score of 0.985. Conversely, when the mutated sequence (containing the Arg89-His90 motif) was analyzed, the tool no longer recognized this position as a potential cleavage site for PC2 (Figure 3b).

All the variants were analyzed using the Human Splicing Finder Pro tool. No effect on splicing was observed for p.Ser94Gly, whereas p.Arg90His, p.Ser94= and p.Ala195= were associated with significant alterations to ESE/ESS motifs, which could impact splicing. The p.Ser97_Gly99del, which consists of a deletion/insertion of nine nucleotides (AGCAGCGGC) coding for the three amino acids Ser-Ser-Gly, causes loss/insertion of three full codons, so there are no changes in the amino acid reading frame. However, the splicing prediction showed a significant alteration to the ESE/ESS motif ratio, as well as the activation of a cryptic acceptor site, which could impact splicing. The bioinformatic analysis of the POMC variants is summarized in Table 3.

## 4. Discussion

Obesity is a complex disease caused by an interaction between environmental factors and polygenic inheritance. Nevertheless, there is extensive evidence of a monogenic contribution [15], which affects the hypothalamic leptin–melanocortin signaling pathway [16]. The main actors in this pathway are the *LEP*, *LEPR* and *MC4R* genes. The *LEP* gene is located on human chromosome 7 at the 7q32.1 band. It encodes a protein that is secreted by white adipocytes into the circulation and binds to the leptin receptor in the brain. This activates downstream signaling pathways that inhibit feeding and promote energy expenditure. The *LEPR* gene is located at 1p31.3 and belongs to the gp130 family of cytokine receptors, which are known to stimulate gene transcription by activating cytosolic STAT proteins. Finally, the *MC4R* gene, located at 18q21.32, plays a crucial role in regulating appetite and energy balance. Pathogenic variants in these genes cause severe early-onset obesity, accompanied by hyperphagia and some endocrine disorders [17]. While variants in the *LEP*, *LEPR* and *MC4R* genes are associated with monogenic obesity, the *FTO* and *BDNF* genes are considered to be susceptibility genes. Variants in these genes are associated with an increased risk of common polygenic obesity, influencing body weight regulation, appetite, and energy balance. For instance, FTO variants are known to modulate food intake and preference [18], while BDNF plays a key role in the hypothalamic signaling pathways that control satiety [19]. Although variants in these genes do not cause severe early-onset obesity by themselves, they contribute to a genetic predisposition that, when combined with environmental factors, increases the likelihood of obesity development. In light of this evidence, our routine diagnostic panel screens selected obese patients for mutations in the *LEP*, *LEPR*, *MC4R*, *BDNF* and *FTO* genes. In the present study, we conducted a more in-depth assessment of the genetic factors involved in obesity by expanding the analysis to include two additional components of the hypothalamic melanocortin system, the *POMC* and *MC3R* genes.

In our cohort of 88 patients, no pathogenic variants were identified in *LEP* or *FTO*, while several known polymorphisms were detected in *LEPR* and *BDNF*, mostly with frequencies comparable to those reported in the general and Italian populations. The frequencies of the rs1137101 polymorphism in the *LEPR* gene and the rs6265 polymorphism in the *BDNF* gene align with those reported in our previous study of another Italian patient cohort [9]. Notably, we also identified the rare variant rs8192466 (c.318C>T, p.Thr2Ile) in the *BDNF* gene. The patient carrying this variant, who also harbored the common polymorphism rs6265, was affected by class III obesity and was treated with tirzepatide, titrated up to a maximum dose of 15 mg/week over a period of 72 weeks. Despite adhering strictly to the treatment schedule and experiencing no significant adverse events, the patient only achieved a 9% reduction in weight. This result is notably inferior to the average weight loss of 15–20% observed in Phase III clinical trials [20,21]. It is hypothesized that this partial response may be influenced by the patient’s genetic background, which could result in reduced efficacy of central appetite regulation. This would specifically attenuate the central action of GLP-1/GIP receptor agonists on hedonic hunger and craving. This case highlights the potential value of genetic screening in predicting therapeutic responses. In the *MC4R* gene, only a limited number of variants were observed, including the previously described p.Ile251Leu variant, which has already been associated with obesity [22].

After excluding patients carrying variants that are known to be associated with obesity, the analysis was extended to include the *MC3R* and *POMC* genes in the remaining subjects. In MC3R, the p.Val44Ile variant was found in a subset of patients with a frequency that is consistent with the population data. Although bioinformatic predictions suggest a largely neutral effect, conflicting in silico results highlight the challenge of definitively classifying the functional relevance of this variant. However, the high frequency of this variant in the general population suggests that it is not a pathogenic variant. We cannot exclude that this patient carries another variant in other genes associated with obesity that are not included in the present study, which may have a stronger influence on the phenotype.

More notably, the *POMC* gene analysis revealed several rare variants as well as the relatively common p.Ser97_Gly99del deletion. It is worth noting that two variants, rs28930368 (c.385C>T; p.Ser94=) and rs2071345 (c.585C>T; p.Ala195=), are rare in the Italian population but are relatively common in other ethnic groups, reaching a peak in Asian populations. The observation of similar differences in allele distribution in populations of different origins is not new. In particular, it has been reported that Asian populations have a higher frequency of allele variants associated with specific body fat distribution [23], as well as a higher incidence of abdominal obesity, also known as the ‘skinny fat’ Asian syndrome [24]. Because these rare *POMC* variants were found in the heterozygous state in adult patients, they do not cause the classic severe autosomal recessive *POMC* deficiency syndrome (which typically presents in early childhood with adrenal insufficiency). Instead, these heterozygous variants likely confer strong genetic susceptibility and an intermediate obesity phenotype due to haploinsufficiency, contributing to the severe weight gain observed in our adult cohort. All the identified *POMC* variants are located within regions that are critical for post-translational processing of the POMC prohormone, including the joining peptide and the β-lipotropin domains, which are essential for the generation of biologically active melanocortin peptides. Bioinformatic analyses suggest that some of these variants may affect splicing regulatory elements, while others may interfere with proteolytic cleavage sites. This suggests that these mutations may play a role in causing disease. Further studies will provide a deeper understanding of the mechanisms and test this hypothesis.

Limitations: Our study has some limitations that should be acknowledged. First, the functional impact of the identified *POMC* and *MC3R* variants was assessed exclusively through in silico bioinformatic tools; therefore, further in vitro functional studies are required to definitively confirm their effects on peptide processing and receptor signaling. Second, while family cascade screening is systematically recommended in our clinical genetic reports to trace genotype–phenotype segregation, the real-world nature of this study limited our ability to perform a family segregation analysis as this strictly depends on the voluntary compliance of the patients’ relatives.

## 5. Conclusions

**Figure 4:**
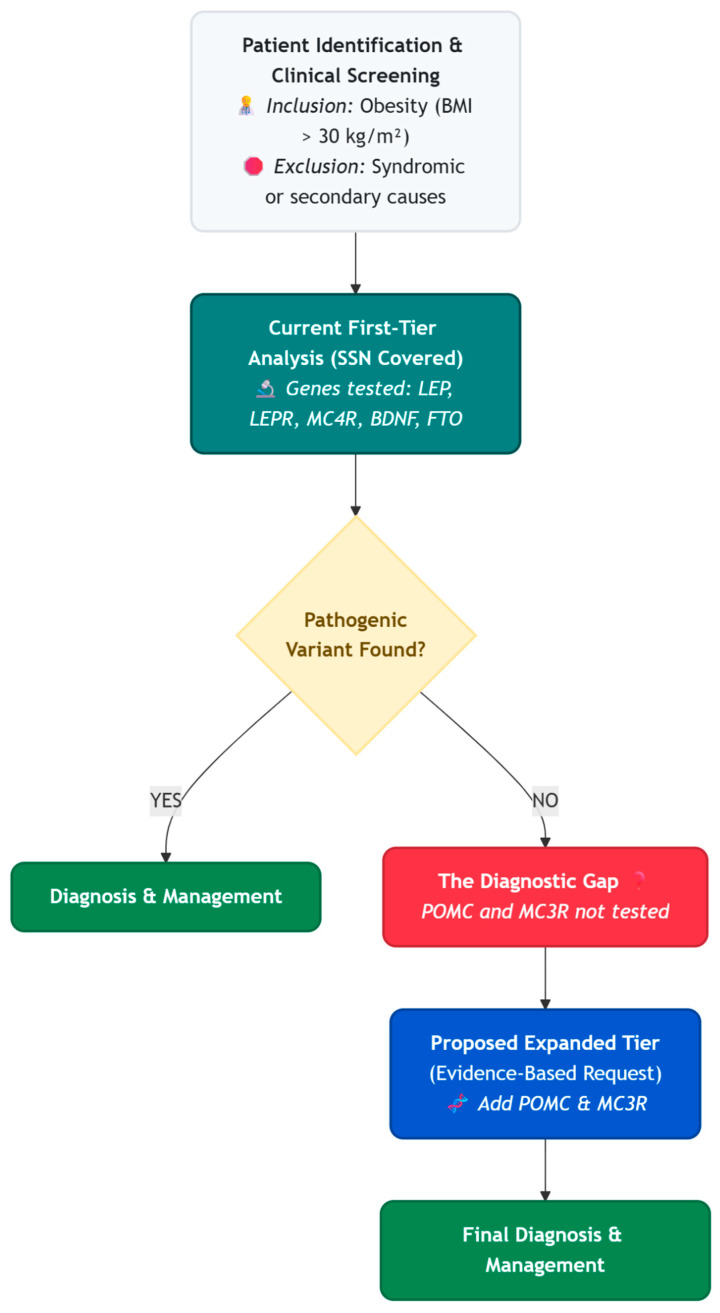
Flowchart illustrating the current clinical practice in our routine first-level analysis and the proposed evidence-based expansion. The current first-tier panel creates a diagnostic gap for patients not tested for POMC and MC3R. We propose to systematically include POMC and MC3R in first-tier requests to optimize patient care.

In this article, we show that the genetic panels typically used in first-level clinical practice may not be sufficient for diagnosing all types of genetic obesity. In light of obesity being recently recognized as a chronic disease in Italy (Law 3 October 2025, n. 149, “Legge Pella”) and included in the Essential Levels of Assistance (LEA), our real-world data provides timely evidence supporting the need to update standard clinical protocols across different regional health systems and expand first-level genetic screenings to universally include *POMC* and *MC3R* (Figure 4). Furthermore, the observation of a suboptimal response to tirzepatide in a patient carrying the *BDNF* p.Thr2Ile variant highlights the potential of pharmacogenomics. This indicates that expanding genetic screening to individuals undergoing pharmacological treatment could be an effective strategy for predicting therapeutic efficacy and personalizing patient care.

## Figures and Tables

**Table 1:** Characteristics of patients with obesity.

Parameter	
Number of subjects (n)	88
Gender (M:F)	24:64
Age at diagnosis (years)	50 ± 12
BMI at diagnosis (kg/m^2^)	46 ± 5.03
Metabolic syndrome (%)	42.10
Steatosis (%)	63
Dyslipidemia (%)	42
Waist circumference (cm)	129.3 ± 12.5
HDL cholesterol (mg/dL)	41.9 ± 10.8
Triglycerides (mg/dL)	189.9 ± 113
Glycemia (mg/dL)	121.2 ± 57.5
Insulin (mUI/L)	26.2 ± 27.4
HOMA-IR	6.9 ± 9.0
Hypertension (%)	47
Steatosis (%)	81
Dyslipidemia (%)	47.2

HOMA-IR: Homeostatic Model Assessment of Insulin Resistance.

**Table 2:** Identified variants in the cohort of 88 patients.

Genes	Variants	Frequency in Our Cohort n° of Patients (%)	Frequency in the General Populations (1000 Genomes Project Phase 3) (%)	Frequency in the Italian Population (1000 Genomes Project Phase 3) (%)
***LEP***	none	/	/	/
***LEPR***	rs1805096 c.3226G>A p.Pro1019=	39 (44.3)	53	37
	rs1137101 c.668A>G p.Gln223Arg	18 (20.5)	58	44
	rs1137100 c.495A>G p.Lys109Arg	27 (30.7)	32	19
***BDNF***	rs6265 c.509G>A p.Val66Met	29 (33.0)	20	24
	rs8192466 c.318C>T p.Thr2Ile	1 (1.1)	<1	0.5
***FTO***	none	/	/	/
***MC4R***	rs52820871 c.751A>C p.Ile251Leu	2 (2.3)	<1	0
	rs1278744683 c.309C>A p.Val103=	1 (1.1)	<1	0
***MC3R***	rs3827103 c.130G>A p.Val44Ile	6 (7.0)	25	8
***POMC***	rs10654394 c.289_297del p.Ser97_Gly99del	11 (12.9)	8	4
	rs1216042661 c.269G>A p.Arg90His	1 (1.2)	<1	0
	rs180767274 c.280A>G p.Ser94Gly	1 (1.2)	<1	<1
	rs28930368 c.385C>T p.Ser94=	1 (1.2)	9	0
	rs2071345 c.585C>T p.Ala195=	1 (1.2)	9	0

**Table 3:** Summary of the bioinformatic analyses applied to *POMC* variants.

	HSF Predicted Impact	PredictSNP	ClinVar	ProsperousPlus
**rs1216042661 c.269G>A p.Arg90His**	Significant alteration of ESE/ESS motif ratio	deleterious	Not reported	Loss of cleavage site
**rs180767274 c.280A>G p.Ser94Gly**	No effect	neutral	uncertain	not applicable
**rs28930368 c.385C>T** **p.Ser94=**	Significant alteration of ESE/ESS motif ratio	not applicable	benign/uncertain	not applicable
**rs2071345 c.585C>T** **p.Ala195=**	Significant alteration of ESE/ESS motif ratio	not applicable	benign	not applicable
**rs10654394 c.289_297del** **p.Ser97_Gly99del**	Significant alteration of ESE/ESS motif ratio; activation of a cryptic acceptor site. Potential alteration of splicing	not applicable	benign/uncertain	not applicable

## Data Availability

The original contributions presented in this study are included in the article/Supplementary Materials. Further inquiries can be directed to the corresponding author.

## Supplementary Materials

The following supporting information can be downloaded at: https://www.mdpi.com/article/10.3390/genes17040405/s1, Supplementary Methods: DNA extraction, PCR, sequencing, and statistical analysis; Table S1. Sequence of primers.
