# Supplementary material for: The Necessity of POMC and MC3R Analysis in the First-Level Diagnosis of Monogenic Obesity: The Experience of Two Italian Centers
Source: Genes (Basel). 2026 Mar 31;17(4):405. doi: 10.3390/genes17040405 (PMC13116659; doi:10.3390/genes17040405)
Supplement: Supplementary file 1 [file genes-17-00405-s001.zip › genes-4195745-supplementary.pdf]

Supplementary Materials

Supplementary Methods

1S. DNA Extraction and PCR

Genomic DNA was extracted from peripheral blood leukocytes using a salting-out technique and stored at −20◦C until PCR analysis. The DNA samples were analyzed by Nanodrop One (Thermo Scientific, Milan, Italy) for concentration and purity. All samples displayed an OR260/280 ratio of ≥1.8. The study of single nucleotide polymorphisms (SNPs) was conducted by PCR amplification using FastStart Taq DNA Polymerase (Roche Diagnostics Deutschland GmbH, Mannheim, Germany). The PCR reaction mixture (25 μl) contained 0.2 μM of each of the two oligonucleotide primers and 100 ng of DNA. The cycling conditions consisted of an initial denaturation and Taq polymerase activation step at 95 °C for 3 minutes, followed by 30 cycles of 1 minute at 94 °C, 1 minute at 60 °C and 1 minute at 72 °C, with a final extension step at 72 °C for 10 minutes. These amplification conditions were set up by performing a gradient of annealing temperatures and verifying the specificity of the PCR products by sequencing.
Specific primers were designed using Primer3 Input tool and purchased from Eurofins Genomics (Ebersberg, Germany). Primers sequences and PCR conditions are reported in the Supplementary Table 1.

2S. Sequencing

The entire set of samples was directly sequenced by Sanger sequencing. In brief, the PCR products were purified using ExoSAP-IT™ PCR Product Cleanup Reagent, after which the sequencing reaction was performed with the BigDye™ Terminator v1.1 Cycle Sequencing Kit. The products were then purified using the BigDye XTerminator™ Purification Kit to remove unincorporated BigDye™ terminators and salts, before being sequenced using the Thermo Fisher 3500 Series Genetic Analyzer (all products provided by Thermo Fisher Scientific, Milan, Italy).

3S. Statistical analysis

A statistical analysis wad performed to compare the frequencies of SNPs present in our cohort of patients to those reported in the general and Italian populations. All statistical analyses were carried out by using the software package SPSS v13.0. A p-value of less than 0.05 was considered statistically significant.Interaction with polymorphisms was tested by $\chi 2$ analysis at genotype and allele levels.

Supplementary table S1. Sequence of primers

**LEP-exon2**	Forward: GCAGTGTGTGGTTCCTTCTG<br>Reverse: GCATTCAGGAGGCGTTCAAT
**LEP-exon3**	Forward: GCACTTGTTCTCCCTCTTCCT<br>Reverse: GTTCCTTCCCTTAACGTAGTCCT
**LEPR-exon1**	Forward: GCGTACAGGAATAAATCTGTAG<br>Reverse: AGGAGGTTGGGAAGGGTTTT
**LEPR-exon2**	Forward: GCACTACATGGTTTAATCTCAGA<br>Reverse: AAAATCATAGCCATAAGACATCT
**LEPR-exon3**	Forward: CGCATGCCACTAGTTAAAGCT<br>Reverse: GGAAATGTAGGGATGCAAGAGG
**LEPR-exon4**	Forward: CCTTTAAGCTGGGTGTCC<br>Reverse: CCCCAGTACTACATCTACCA
**LEPR-exon5**	Forward: ACGAGACCCAGTATTCAAGCT<br>Reverse: TGCTATGGGACTTAAGAGGGTC
**LEPR-exon6**	Forward: AAGGTTCCACATCAACTTGA<br>Reverse: AGAAACACACAAGCCTAGAG
**LEPR-exon7**	Forward: AACTCTGGAATGTGTTGTGA<br>Reverse: CCTCTGTTTTCTTACCAATCAC
**LEPR-exon8**	Forward: CTGCAATGAACATGAATGCC<br>Reverse: TTTTATCTCACTGTGCCCAC
**LEPR-exon9**	Forward: GTCTTCCATGAAACCGGTCC<br>Reverse: GAAGACACAACGCAGCTTGA
**LEPR-exon10**	Forward: TTGCTTGATGAATACAGATGT<br>Reverse: AGCTACACAATACAGGAGAA
**LEPR-exon11**	Forward: GGCAGAGAACACAGAATCAGT<br>Reverse: TGTGTGCTTCAAATATGGCTGA
**LEPR-exon12**	Forward: TGTGTGCTTCAAATATGGCTGA<br>Reverse: CAGGATTGTTGAGCTTTCCGA
**LEPR-exon13**	Forward: AATAGTACCTGCCCTGATCT<br>Reverse: ACCACTCTGTACCTCTTCTT
**LEPR-exon14**	Forward: CTGTCTTCTCTTCCTTATTCCCT<br>Reverse: ATCCATGAGAGCAACTTACTAAA
**LEPR-exon15**	Forward: GCAATCTTCTGTCTCTGGCTAA<br>Reverse: GCAAACAAATTAGGCACACACA
**LEPR-exon16**	Forward: CAGGTAGAGAGGAATGGCAAAT<br>Reverse: TGCCGTAAAGATTCTTAACCTCA
**LEPR-exon17A**	Forward: AGGCATAGTTGATCTGGTGGA<br>Reverse: TCATACGATAAACTATGCGCTGA
**LEPR-exon17B**	Forward: TGCAATCTAGACGCCATATGAC<br>Reverse: TGTACTTGAATGGAACTTCTGCA
**LEPR-exon18A**	Forward: TCCATTTCTGCCAGTATGACA<br>Reverse: TTGTCTCTGGCTTTCGTCCT
**LEPR-exon18B**	Forward: AGGCTGAGGGTACTGAGGTA<br>Reverse: AGCACACCACTCTCTCTCTT

**LEPR-exon18C**	Forward: TCACCACACCTCACATTCTCA<br>Reverse: TTCTCTCTCCCACCCACAAC
**BDNF-exon1A**	Forward: CCGGTGAAAGAAAGCCCTA<br>Reverse: AGAAGAGGAGGCTCCAAAGG
**BDNF-exon1B**	Forward: TGTACACGTCCAGGGTGATG<br>Reverse: ATGGGATTGCACTTGGTCTC
**BDNF-exon1C**	Forward: GACGGTCACAGTCCTTGAAAA<br>Reverse: TGTTTCCCTTCTGGTCATGG
**FTO-exon1**	Forward: CAGGACGCTGAGAGAACTACA<br>Reverse: CTTCCGGTTCCCTGATCCTT
**FTO-exon2**	Forward: TCAAAGTTGGCTAAAATTTGTTTG<br>Reverse: AGGTTGATCAGAACACATCACTG
**FTO-exon3A**	Forward: GGGCTAGGAAGATGTGACTCC<br>Reverse: CCTTCTCTTTGGCAGCAAGT
**FTO-exon3B**	Forward: GGAAGAACTTGCTGCCAAAG<br>Reverse: AGCATGGCCAGATGAGAAAG
**FTO-exon4**	Forward: TTTTAAAAATATGACATAAAGGGAAGA<br>Reverse: GGGCAACAAGAGTGAAGCTC
**FTO-exon5**	Forward: TCCTTGGTTATTGTTATTTCTTGGA<br>Reverse: GGAAGCAATTTTCCCATCAA
**FTO-exon6**	Forward: AATTCACAGCCAGGGACAAA<br>Reverse: CAAAATCAGCCAGGAGTGGT
**FTO-exon7**	Forward: CCTTTCTCACCTTTTCTCATCC<br>Reverse: TCCTGGCTATACCCATCACC
**FTO-exon8**	Forward: ATATATGCCGGCCATTTTTG<br>Reverse: CCCTCAAAGGAAAGGGATGA
**FTO-exon9**	Forward: CCTGCGTTCCACCTGTAGAT<br>Reverse: AACTTCATGGGCACCATTTC
**MC4R-exon1A**	Forward: GCAGGCATGGCAATTTTAGC<br>Reverse: TGTGAAACTCTGTGCATCCG
**MC4R-exon1B**	Forward: TGTGGCTGATATGCTGGTGA<br>Reverse: CGCTCCCTTCATATTGGCAC
**MC4R-exon1C**	Forward: GTCCACATGTTCCTGATGGC<br>Reverse: TCAACCAGTACCCTACACGG
**MC3R-exon1A**	Forward: CTGTCTCTCTACCCTCCCC<br>Reverse: AACCTCGGGCTTGATGAAGA
**MC3R-exon1B**	Forward: CAGCCAACACTGCCTAATGG<br>Reverse: AGCGCGTAAAAGATGGTGAC
**MC3R-exon1C**	Forward: GGTAAGTGTGTCCAATGCCC<br>Reverse: GAATGGTGATGGTGACTGCC
**MC3R-exon1D**	Forward: GGCAGTCACCATCACCATTC<br>Reverse: ACCTCACGTGGATGGAAAGT
**POMC-exon2***	Forward: GGTGAGTGGCCAACATTGTT<br>Reverse: CCAGCTCCAGTCCCATCTAA
**POMC-exon3A**	Forward: ACTGTGCCCTGTGTCCTC<br>Reverse: GTCTTCGCCCGCTGAGAC
**POMC-exon3B**	Forward: AAGTACGTCATGGGCCACTT<br>Reverse: CAGCTCCCTCTTGAACTCCA

**POMC-exon3C**	Forward: CGCCCAGTGAAGGTGTACC<br>Reverse: TTTTGAACAGCGTCACCAGG
**POMC-exon3D**	Forward: GGCCGAGAAGAAGGACGAG<br>Reverse: TACAGGCAGCTTTAAGAGGCT

* Translation starts from exon2
